# Modulation of Mononuclear Phagocyte Inflammatory Response by Liposome-Encapsulated Voltage Gated Sodium Channel Inhibitor Ameliorates Myocardial Ischemia/Reperfusion Injury in Rats

**DOI:** 10.1371/journal.pone.0074390

**Published:** 2013-09-19

**Authors:** Xin Zhou, Yue-Chen Luo, Wen-Jie Ji, Li Zhang, Yan Dong, Lan Ge, Rui-Yi Lu, Hai-Ying Sun, Zao-Zeng Guo, Guo-Hong Yang, Tie-Min Jiang, Yu-Ming Li

**Affiliations:** 1 Institute of Cardiovascular Disease and Heart Center, Pingjin Hospital, Logistics University of the Chinese People’s Armed Police Forces, Tianjin, China; 2 Tianjin Key Laboratory of Cardiovascular Remodeling and Target Organ Injury, Tianjin, China; 3 Department of Pharmacognosy and Pharmaceutics, Logistics University of the Chinese People’s Armed Police Forces, Tianjin, China; Northwestern University Feinberg School of Medicine, United States of America

## Abstract

**Background:**

Emerging evidence shows that anti-inflammatory strategies targeting inflammatory monocyte subset could reduce excessive inflammation and improve cardiovascular outcomes. Functional expression of voltage-gated sodium channels (VGSCs) have been demonstrated in monocytes and macrophages. We hypothesized that mononuclear phagocyte VGSCs are a target for monocyte/macrophage phenotypic switch, and liposome mediated inhibition of mononuclear phagocyte VGSC may attenuate myocardial ischemia/reperfusion (I/R) injury and improve post-infarction left ventricular remodeling.

**Methodology/Principal Findings:**

Thin film dispersion method was used to prepare phenytoin (PHT, a non-selective VGSC inhibitor) entrapped liposomes. Pharmacokinetic study revealed that the distribution and elimination half-life of PHT entrapped liposomes were shorter than those of free PHT, indicating a rapid uptake by mononuclear phagocytes after intravenous injection. In rat peritoneal macrophages, several VGSC α subunits (NaV1.1, NaV1.3, NaV1.4, NaV1.5, NaV1.6, NaV1.7, NaVX, Scn1b, Scn3b and Scn4b) and β subunits were expressed at mRNA level, and PHT could suppress lipopolysaccharide induced M1 polarization (decreased TNF-α and CCL5 expression) and facilitate interleukin-4 induced M2 polarization (increased Arg1 and TGF-β1 expression). In vivo study using rat model of myocardial I/R injury, demonstrated that PHT entrapped liposome could partially suppress I/R injury induced CD43+ inflammatory monocyte expansion, along with decreased infarct size and left ventricular fibrosis. Transthoracic echocardiography and invasive hemodynamic analysis revealed that PHT entrapped liposome treatment could attenuate left ventricular structural and functional remodeling, as shown by increased ejection fraction, reduced end-systolic and end-diastolic volume, as well as an amelioration of left ventricular systolic (+*dP/dt*_max_) and diastolic (-*dP*/*dt*_min_) functions.

**Conclusions/Significance:**

Our work for the first time demonstrates the therapeutic potential of VGSC antagonism via liposome mediated monocyte/macrophage targeting in acute phase after myocardial I/R injury. These results suggest that VGSCs in mononuclear phagocyte system might be a novel target for immunomodulation and treatment of myocardial I/R injury.

## Introduction

Inflammatory response plays an important role in the healing process and pathological left ventricular remodeling after myocardial ischemia/reperfusion (I/R) injury [1,2]. Mounting evidence shows that inhibition of excessive inflammation could limit the infarct size and ameliorate heart function [3-5]. Mononuclear phagocytes (monocytes and macrophages) are the major inflammatory cells that stay for the longest duration in the ischemic region following myocardial infarction. It is generally acknowledged that mononuclear phagocytes are a heterogeneous population: the inflammatory mononuclear phagocytes have a strong capacity of phagocytosis and chemotaxis; the anti-inflammatory mononuclear phagocytes could induce angiogenesis and extracellular matrix remodeling, thus promoting tissue repair [6,7]. Recent basic studies have provided ample evidence indicating that anti-inflammatory strategies targeting murine inflammatory monocyte subset could reduce excessive inflammation and improve cardiovascular outcomes [4,8-11].

Voltage-gated sodium channels (VGSCs/NaV) are essential components involved in action potential formation and propagation in myocardiocytes, skeletal muscle cells and neurons [12-14]. In addition to these excitable cells, functional expression of VGSCs has been reported in many non-excitable cells, such as tumor cells [15,16], T cells [17,18], as well as bone marrow-derived monocytes/macrophages [19-21]. Tetrodotoxin (TTX), a selective VGSCs antagonist, inhibits phagocytosis of lipopolysaccharide (LPS)-stimulated THP-1 cells and human monocyte-derived macrophages through the blockade of intracellular VGSCs (located on endosomes and podosomes), whereas VGSC agonist veratridine could exert the opposite effects [19,20]. These results suggest that the VGSCs would be a novel target that involved in macrophage polarization or phenotype switch.

Phenytoin (PHT) is a widely used anticonvulsant and antiarrhythmic drug, which non-selectively blocks VGSCs. The commonly encountered side effect of this drug is gingival hyperplasia, which may be due to the local activation of tissue macrophage by PHT [22]. Given that macrophage plays an important role in wound healing, further studies take the advantage of PHT’s side effect by showing that tropic use of PHT could accelerate the healing of various types of soft tissue wounds [23,24]. Enlightened by these findings, our previous work demonstrated that intraperitoneal administration of PHT could facilitate the post-infarction healing process in a rat model of permanent coronary ligation [25]. It remains unclear whether or not PHT’s effect on wound healing is dependent on VGSCs in mononuclear phagocytes. Liposome is a commonly used vehicle in the field of pharmaceutics and molecular biology. Liposome is engulfed by the mononuclear phagocyte system via opsonization after intravenous injection and is prone to recruit into the sites of inflammation due to increased vascular permeability [26]. These advantages make liposome an ideal tool for mononuclear phagocyte targeted therapy. Therefore, in this work, we hypothesized that VGSCs is a target for monocyte/macrophage phenotypic switch, and liposome mediated inhibition of mononuclear phagocyte VGSC may attenuate myocardial I/R injury and improve post-infarction left ventricular remodeling.

## Materials and Methods

### Animals

Male Wistar rats (weighing 200-250 g, 8 to 10 weeks old) were purchased from the Laboratory Animal Center of The Academy of Military Medical Sciences (Beijing, China). Animals received humane care in compliance with the Regulations for Management of Experimental Animals (Tianjin Municipal Science and Technology Commission, revised June 2004) which was in accordance with Guide for the Care and Use of Laboratory Animals published by the National Institutes of Health (NIH Pub. no. 85-23, revised 1996). All experimental procedures were performed with the authorization of the Animal Use and Care Committee of the Logistics University of the Chinese People’s Armed Police Forces.

### Preparation and characterization of PHT entrapped liposomes

The liposomes were prepared from a lipid mixture of soybean phosphatidylcholine and cholesterol at 6: 1 mass ratios by standard thin film dispersion method as previously described [27]. The Zeta potential and particle size of liposome were detected by Zeta plus particle size analyzer (Malvern, England). Micrographs were taken on a transmission electron microscope (JEOL, Japan).

### Estimation of pharmacokinetic characteristics of PHT entrapped liposomes by high performance liquid chromatography (HPLC)

Rats were randomly divided into two groups: 1) PHT (10 mg/kg, n=5); 2) PHT-lipo (10 mg/kg for entrapped PHT, n=5). Drugs were given by tail vein injection. Blood samples were collected from the submandibular vein at 5, 10, 30, 60, 90, 120, and 210 min post-injection. Plasma was separated from the whole blood. The PHT concentrations in rat plasma were analyzed by a HPLC system (LC-20AT, Shimadzu, Japan), with a SHIM-PACK VP-ODS C18 (150 mm × 4.6 mm, 5 µm) column at 30 °C. The mobile phase was consisted of methanol-water-phosphoric acid (80: 20: 0.4). The flow rate was 1 mL/min and detection wavelength was fixed at 240 nm. The entrapment efficiency was also determined by HPLC.

### Isolation and culture of rat peritoneal macrophages

The rats were sacrificed under ether anesthesia and the peritoneum was washed three times with cold DMEM (HyClone, USA) in order to collect cell lavage. Peritoneum lavage was centrifuged (1000 rpm, 4 °C, 10 min) and the pellet was resuspended in a culture medium comprised of DMEM, supplemented with 2 mM L-glutamine (Amresco, USA), 10% fetal bovine serum (FBS, HyClone, USA) and plated to select for the adherent macrophages.

### Isolation of rat dorsal root ganglion (DRG)

The rats were sacrificed under ether anesthesia and the fur is disinfected by spraying with 75% ethanol. The prepared animal was placed in the prone position; then a posterior midline incision was made. The spinal column was exposed with blunt separation. Following operation was visualized under the operation microscope (Zeiss, German). To expose DRG, the lamina of vertebra and transverse were removed with rongeur and spinal cord was lifted with tweezers. The DRG was isolated with dissecting scissors and stored in liquid nitrogen for total RNA extraction.

### VGSC mRNA expression in rat peritoneal macrophages and DRG

The mRNA expression level of VGSCs was quantified by reverse transcriptase polymerase chain reaction (RT-PCR). Total RNA was extracted using Trizol reagent (Invitrogen, USA), and cDNA was synthesized from 2 µg of total RNA using M-MLV reverse transcriptase (Promega, USA). PCR was carried out by GeneAmp PCR System 2400 (PerkinElmer, USA). The primer sequences are shown in Table **1**. The PCR products were analyzed by gel electrophoresis and visualized using Chemi-Doc^TM^ XRS (Bio-Rad, USA).

**Table 1:** Primer sequences used in PCR analysis.

**Gene**	**Forward primer**	**Reverse primer**	**Expected PCR product (bp**)
NaV1.1	CGTATGTTCTGCCCCTGTCA	AGGAACAACATCTCCCGAGC	120
NaV1.2	ACCTGCACTGGAGACTGCTA	CACTGTTTAGCTCCTCGCGT	102
NaV1.3	ACAGGAAGCGGAGGCATAAG	TGGTTAGAATGGACGGTGCC	155
NaV1.4	TGAACCCAGGAGACAGTTCG	ATAGATTGTGCCACCTTCGGG	105
NaV1.5	CTGACTATAGCCGCAGCGAA	GGCTCATCTGCAGAGCTAGG	175
NaV1.6	GGCAAACATCGAGAGGCGTA	CACTGTTTGGCTTGGGCTTG	107
NaV1.7	AAAACAGTCCCTTGCCCTCAT	GCTCTGACACCATTCCAGGG	173
NaV1.8	CTGAGCCCACCTCATGATCC	ATTGGTAGTTCCCACGGACG	156
NaV1.9	ATACGGTGCCCTGATCCTCT	GGAAGTGAAGGGGCGGAAAT	105
NaVX	CTTGTCCCATTCACGGCAGA	CTTCCAAGGGCTCTGACACG	175
Scn1b	CCTGCTTTTGCTGCCTGTTT	CCCCTCTTCACCCCATCAAG	148
Scn2b	CCTTGGTCCCTCAATCACCC	ACTGTGACTTCCATGCTCCG	161
Scn3b	CAGGTGTCCCAACACATCCA	CTCTTCGGTTCAGGCAAGGT	120
Scn4b	ACGTCTGACCGACCAGTTTC	CGAGATCCAACACACCACCA	107
TNF-α	ATACACTGGCCCGAGGCAAC	CCACATCTCGGATCATGCTTTC	75
CCL5	CCATATGGCTCGGACACCACTC	CACTTCTTCTCTGGGTTGGCAC	167
TGF-β1	AAACGGAAGCGCATCGAA	GGGACTGGCGAGCCTTAGTT	63
Arg1	GTCTCCAGATGCCTTTGCTTC	ATGAAATTCAGGGTGTGGGAAT	184
β-actin	TCTGTGTGGATTGGTGGCTCT	AGAAGCATTTGCGGTGCAC	115

### Expression of phenotypic markers in differentially activated rat peritoneal macrophages

Rat peritoneal macrophages were treated with different stimuli. 1) LPS (20 ng/mL); 2) LPS+PHT (20 µmol/mL); 3) LPS+PHT (40 µmol/mL); 4) interleukin-4 (IL-4, 20 ng/mL); 5) IL-4+PHT (20 µmol/mL); 6) IL-4+PHT (40 µmol/mL). 24 hours later, total RNA was extracted for real-time quantitative PCR (real-time qPCR) analysis by using an ABI Prism 7300 system (Applied Biosystems, USA). Annealing and elongation were performed at 60°C for 1 min for all genes. Markers used as M1 polarization were tumor necrosis factor-α (TNF-α) and c-c chemokine ligand 5 (CCL5); For M2 polarization, arginase 1 (Arg1) and transforming growth factor-β1 (TGF-β1) were used. The primer sequences are shown in Table **1**. Relative expression levels were normalized with β-actin and calculated with the 2^-ΔΔCt^ method [28].

### Rat model of myocardial I/R injury and treatment protocol

Myocardial I/R injury was induced as previously described in our lab [29]. Briefly, rats were anesthetized with pentobarbital sodium (40 mg/kg ip) and submitted to artificial ventilation (75 strokes/min, tidal volume 8 mL/kg) using a volume-controlled rodent respirator (Harvard Apparatus, USA). Subsequently, a thoracotomy through the left 4^th^ intercostal space was performed, and the heart was exposed. The left anterior descending (LAD) was ligated for 45 min with an intramural 5-0 silk suture, followed by persistent reperfusion. Then chest was then closed, and rats were allowed to recover. The sham groups underwent the same surgical procedure except that the LAD was not occluded. Surviving rats were randomly divided into 6 groups: 1) Sham operated rats treated with saline (Sham-Saline, n=7); 2) Sham treated with empty liposome (Sham-Emp-lipo, n=6); 3) Sham treated with phenytoin-entrapped liposome (Sham-PHT-lipo, n=6); 4) MI treated with saline (MI-Saline, n=7); 5) MI treated with empty liposome (MI-Emp-lipo, n=9); 6) MI treated with phenytoin entrapped liposome (MI-PHT-lipo, n=10). Saline (500 µL), Emp-lipo (500 µL) or PHT-lipo (10 mg/kg, 500 µL) was administrated via tail vein immediately and on day 2 and day 4 day after surgery.

### Flow cytometry analysis of circulating monocyte subsets

For flow cytometry analysis, ethylenediamine tetra-acetic acid (EDTA) anticoagulated blood samples (100 µL) were taken from tail vein at baseline (one day before surgery) and on day 1, 3, 5, 7, 14 and 30 after surgery. 50 µL whole blood samples were stained to identify monocyte subsets as previously described [30]. Samples were washed with phosphate buffered saline (PBS) and subsequently incubated with anti-CD172a (Biolegend, USA) and phycoerythrin (PE)-conjugated anti-CD43 (Biolegend, USA) for 10 min in the dark at room temperature. Then washed with PBS and incubated with anti-IgG-PerCP-Cy5.5 for 10 min in the dark at room temperature. After antibody incubation, 1 mL red blood cell lysis buffer was added and the solution was incubated for 10 min. Data of prepared samples were acquired using a Cytomics FC500 cytometer (Beckman-Coulter, USA) and analyzed using FlowJo software (Treestar, USA). At least 150, 000 events were collected in each sample. Mononuclear cells were first selected from the forward/side scatter plot [31]. Subsequently the monocytes were selected as the CD172a positive cells. Then, two monocytes subsets were identified: CD43+ (classical subset) and CD43++ (non-classical subset).

### Echocardiography

Echocardiographic measurements were done on day 30 post operation as previously described [32]. The rats were anesthetized by intraperitoneal injection of pentobarbital sodium solution (40 mg/kg body weight), then transthoracic echocardiography of the left ventricle was performed using a system equipped with a 12.0 MHz transducer (S12-4 scanner and iE33 xMATRIX ultrasound system, Philips, USA). Two-dimensional B-Mode echocardiograms were captured from parasternal long-axis views as well as mid-papillary short-axis views of the left ventricle. Left ventricular end-diastolic volume (EDV), and left ventricular end-systolic volume (ESV), as well as the left ventricular internal diameters at end-diastole (LVIDD) and at end-systole (LVIDS) were all measured. The percentage of left ventricular ejection fraction (EF) and fractional shortening (FS) were calculated to assess the left ventricular systolic function. All echocardiography examinations were made off-line by a skilled observer blinded to the treated group.

### Invasive hemodynamic measurements

After echocardiographic measurement, the rats were anesthetized with intraperitoneal pentobarbital sodium (40 mg/kg) for invasive hemodynamics using a 2F high fidelity microtip pressure catheter (SPR-320, Millar Instruments, USA) to measure systolic and diastolic blood pressures (SBP and DBP), left ventricular end diastolic pressure (LVEDP), left ventricular end systolic pressure (LVESP), maximal slope of systolic pressure increment (+*dP/dt*_max_), and maximal slope of diastolic pressure decrement (-*dP*/*dt*_min_) as previously described [29,32].

### Histological analysis

After invasive hemodynamic analysis, rats were anesthetized with intraperitoneal pentobarbital sodium (40 mg/kg), and perfused with pre-cooled PBS via the inferior vena cava for 5 min. The hearts were quickly removed, and were prepared for paraffin embedding, sectioned into 5 µm slices. Capillary density was evaluated using fluorescein isothiocyanate (FITC)-labeled isolectin B4 (endothelial cell marker; Sigma Aldrich, USA) staining. Nuclei were stained with 4, 6-diamidino-2-phenylindole (DAPI; Sigma Aldrich, USA). The mean cardiomyocyte cross-sectional area in the left ventricular septum was stained with tetramethylrhodamine isothiocyanate (TRITC)-labeled wheat germ agglutinin (WGA; Invitrogen, USA). Infarct size, index of expansion and collagen volume fraction (CVF) were analyzed using Masson's trichrome stained sections as previously described [33]. All image analyses were performed with Nikon microscopes (80i and E600POL, Nikon, Japan) using a Microsoft Windows-based image analysis software, Image Pro Plus version 4.5 (Media Cybernetics, USA).

### Statistical analysis

Data were presented as the mean ± SEM, and evaluated by either a Student’s unpaired t-test, analysis of variance (ANOVA), or a two-way ANOVA. A two tailed *P*<0.05 was considered statistically significant. All statistical analyses were performed by using Graphpad Prism software (GraphPad Software, USA). The pharmacokinetic parameters were estimated using Kinetica 4.4 (Thermo, USA).

## Results

### Characterization of PHT entrapped liposomes

**Figure 1:**
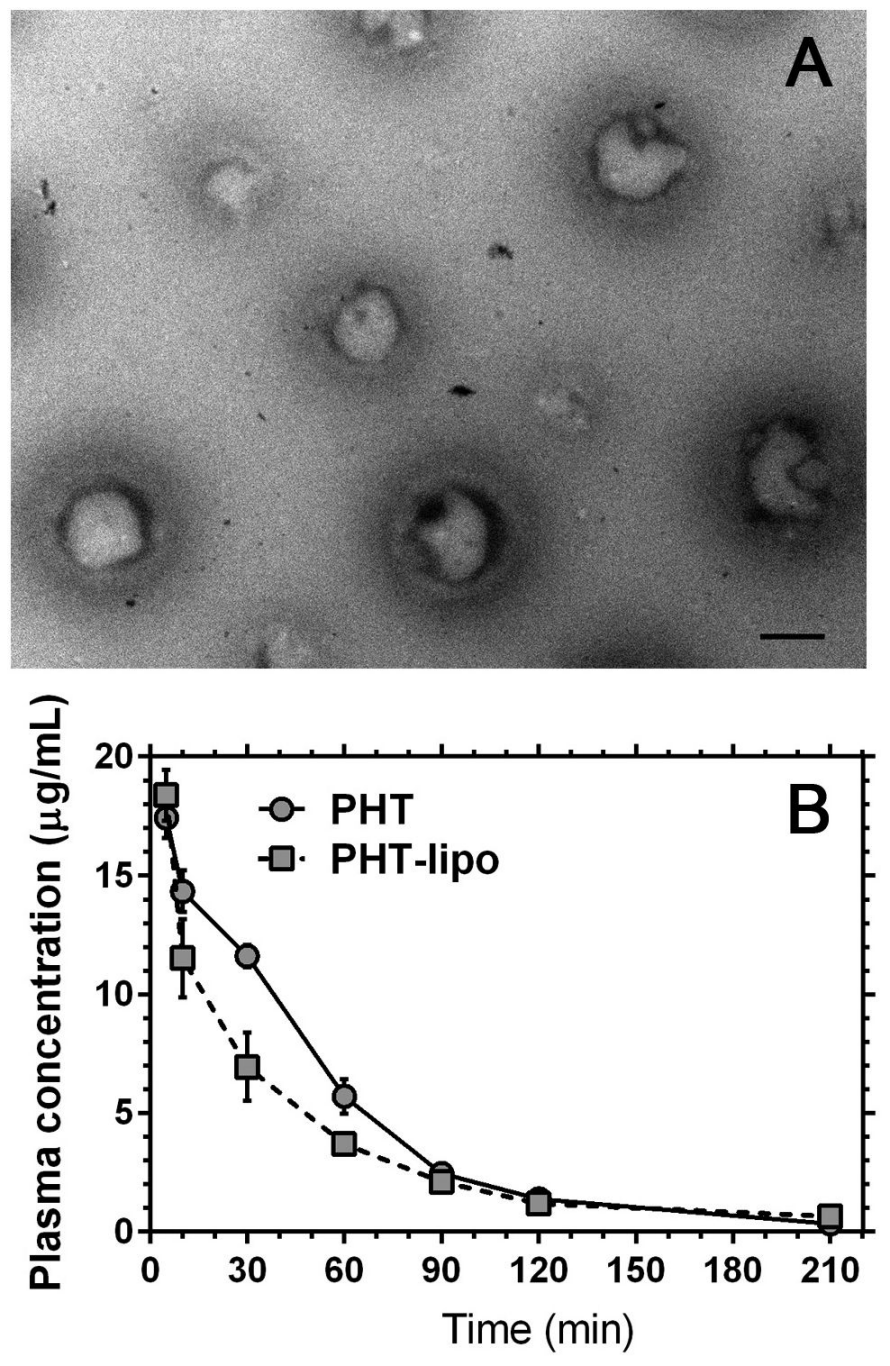
Liposome characterization. (A) Transmission electron microscope image of phenytoin entrapped liposomes. Bar=100 nm. (B) Pharmacokinetics of phenytoin entrapped liposome and free phenytoin in rats following an intravenous injection dose of 10 mg/kg.

The Zeta potential of PHT-lipo was -15.9 mV. The liposomes had an average particle size of 131.5 nm for efficient uptake by mononuclear phagocyte system. The PHT-lipo was multi-lamella (Figure **1A**). The concentration-time curves of PHT and PHT-lipo fitted two-compartment model in rats (Figure **1B**). The distribution half-life (T_1/2_α) and elimination half-life (T_1/2_β), as well as the area under the time concentration curve (AUC) of PHT-lipo were shorter than those of free PHT, indicating a rapid uptake by mononuclear phagocytes after intravenous injection (Table **2**).

**Table 2:** The pharmacokinetic parameters of phenytoin (PHT) and phenytoin entrapped liposomes (PHT-lipo) after intravenous injection to rats.

**Parameters**	**PHT**	**PHT-lipo**
T_1/2_α (min)	26.35	9.98
T_1/2_β (min)	58.09	46.15
AUC (μg/mL/min)	919.5	697.2
K_12_ (min^-1^)	0.0021	0.0196
K_21_ (min^-1^)	0.0146	0.0355
A (μg/mL)	16.21	12.82
α (min^-1^)	0.0263	0.0695
B (μg/mL)	3.6204	7.7000
β (min^-1^)	0.0119	0.0150

T_1/2_α: α-phase (distribution) half-life; T_1/2_β: β-phase (elimination) half-life; AUC: area under curve; K_12_: rate constant of drug transport from central compartment to peripheral compartment; K21: rate constant of drug transport from peripheral compartment to central compartment; A: zero time intercept of α-phase; α: rate constant of quick disposition; B: zero time intercept of β-phase; β: rate constant of slow disposition

### VGSC mRNA expression in rat peritoneal macrophages

**Figure 2:**
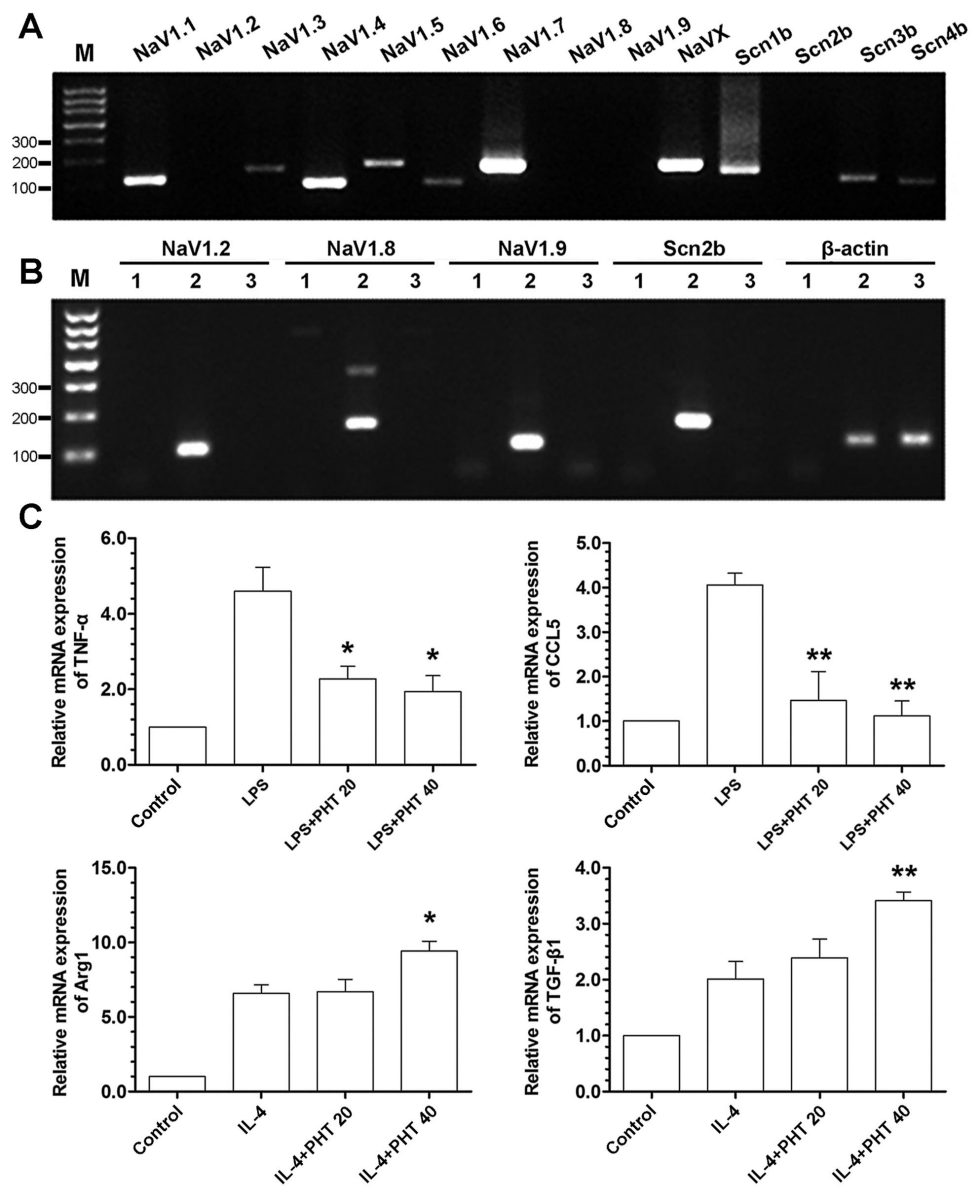
RT-PCR analysis of VGSC expression in rat peritoneal macrophages (A) and dorsal root ganglion (B), and the effect of VGSC antagonism on macrophage polarization (markers were determined by real-time qPCR). (A) Agarose gel electrophoresis of PCR products of VGSCs in rat peritoneal macrophages. (B) Agarose gel electrophoresis of PCR products of VGSCs in rat dorsal root ganglion. Land 1 contains no template; Lane 2 contains sample from dorsal root ganglion; Lane 3 contains samples from rat peritoneal macrophages. (C) The mRNA expression levels of phenotypic markers in classically activated (induced by LPS) and alternatively activated (induced by IL-4) rat peritoneal macrophages by real-time qPCR. PHT 20 indicates phenytoin concentration at 20 µmol/mL, and PHT 40 indicates 40 µmol/mL. ^*^ *P*<0.05 vs. control, ^**^ *P*<0.01 vs. control.

As shown in Figure **2A**, NaV1.1, NaV1.3, NaV1.4, NaV1.5, NaV1.6, NaV1.7, NaVX, Scn1b, Scn3b and Scn4b were expressed in rat peritoneal macrophages. The expression of NaV1.2, NaV1.8, NaV1.9 and Scn2b mRNA were confirmed in rat DRG (Figure **2B**), thus excluding the possibility of false-negative results of these four genes in rat peritoneal macrophages.

### PHT regulates macrophage polarization induced by LPS and IL-4

As shown in Figure **2C**, in vitro studies using rat peritoneal macrophages demonstrated that PHT at concentrations of 20 µmol/mL and 40 µmol/mL, could suppress LPS induced M1 polarization (decreased TNF-α and CCL5 expression) and facilitate M2 marker expression (increased Arg1 and TGF-β1 expression) in IL-4 induced M2 polarization.

### PHT-lipo partially suppresses myocardial I/R injury induced CD43+ monocyte expansion

**Figure 3:**
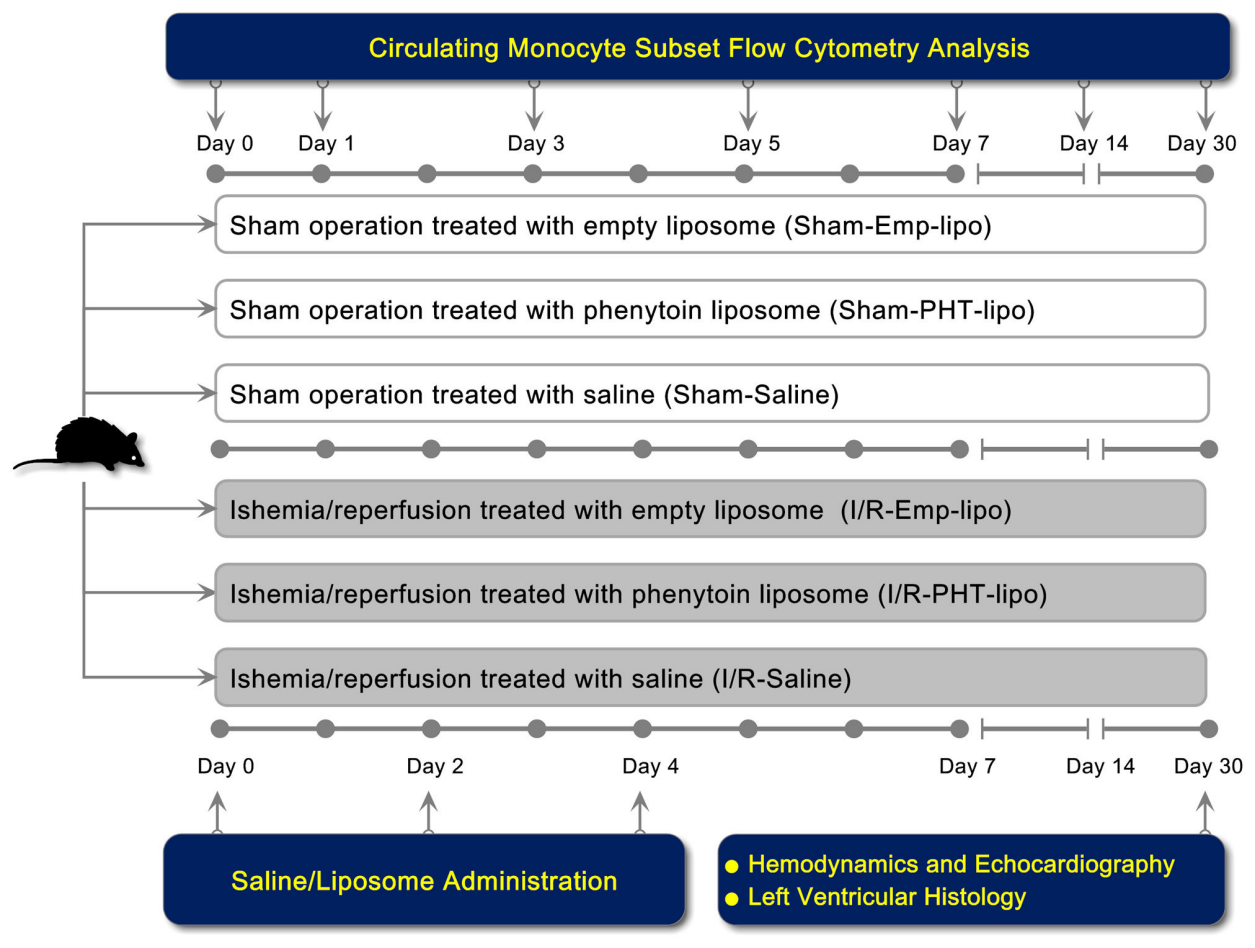
Diagram of in vivo experimental design.

**Figure 4:**
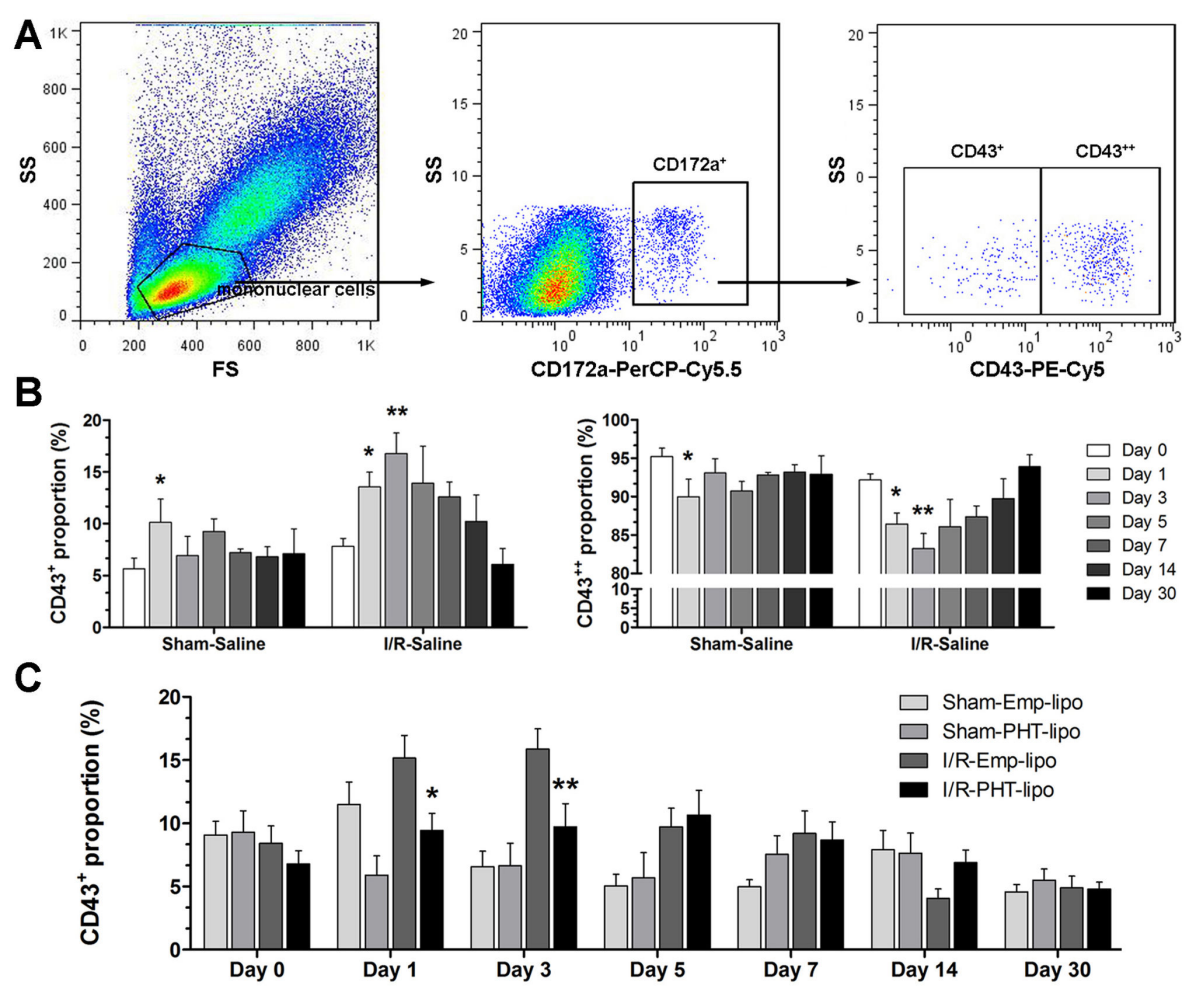
Flow cytometry analysis of rat circulating monocyte subsets. (A) The gating strategy used for analysis of the two monocyte subsets in rat blood. (B) Statistical comparisons of monocyte subset percentages in Sham-Saline and I/R-Saline groups. *P<0.05 *vs* day 0 in the same group. **P<0.01 *vs* day 0 in the same group. (C) Statistical comparisons of CD43+ monocyte subset. *P<0.05 vs. I/R-Emp-lipo group, **P<0.01 vs. I/R-Emp-lipo group.

Figure **3** shows the detailed in vivo experimental design. During the drug intervention study, there was no obvious difference in activity change across all myocardial I/R groups. At the time of sacrifice (day 30), the body weight was comparable in all three myocardial I/R groups (data not shown). Because venous administration of liposome could be quickly engulfed by mononuclear phagocyte system, especially monocytes, we thus evaluated the effect of PHT-lipo on monocyte subset dynamics after myocardial I/R injury. The gating strategy of rat circulating monocyte subset is shown in Figure **4A**. In I/R-Saline group, the proportion of CD43+ monocyte increased on day 1 after surgery compared with baseline, peaked on day 3, and then followed by a gradual decreasing trend till day 30 (Figure **4B**). The dynamics of CD43++ monocytes presented with the opposite pattern (Figure **4B**). As shown in Figure **4C**, PHT-lipo administration significantly suppressed myocardial I/R injury induced CD43+ monocyte pool expansion.

### PHT-lipo attenuates the post-infarction morphological left ventricular remodeling

**Figure 5:**
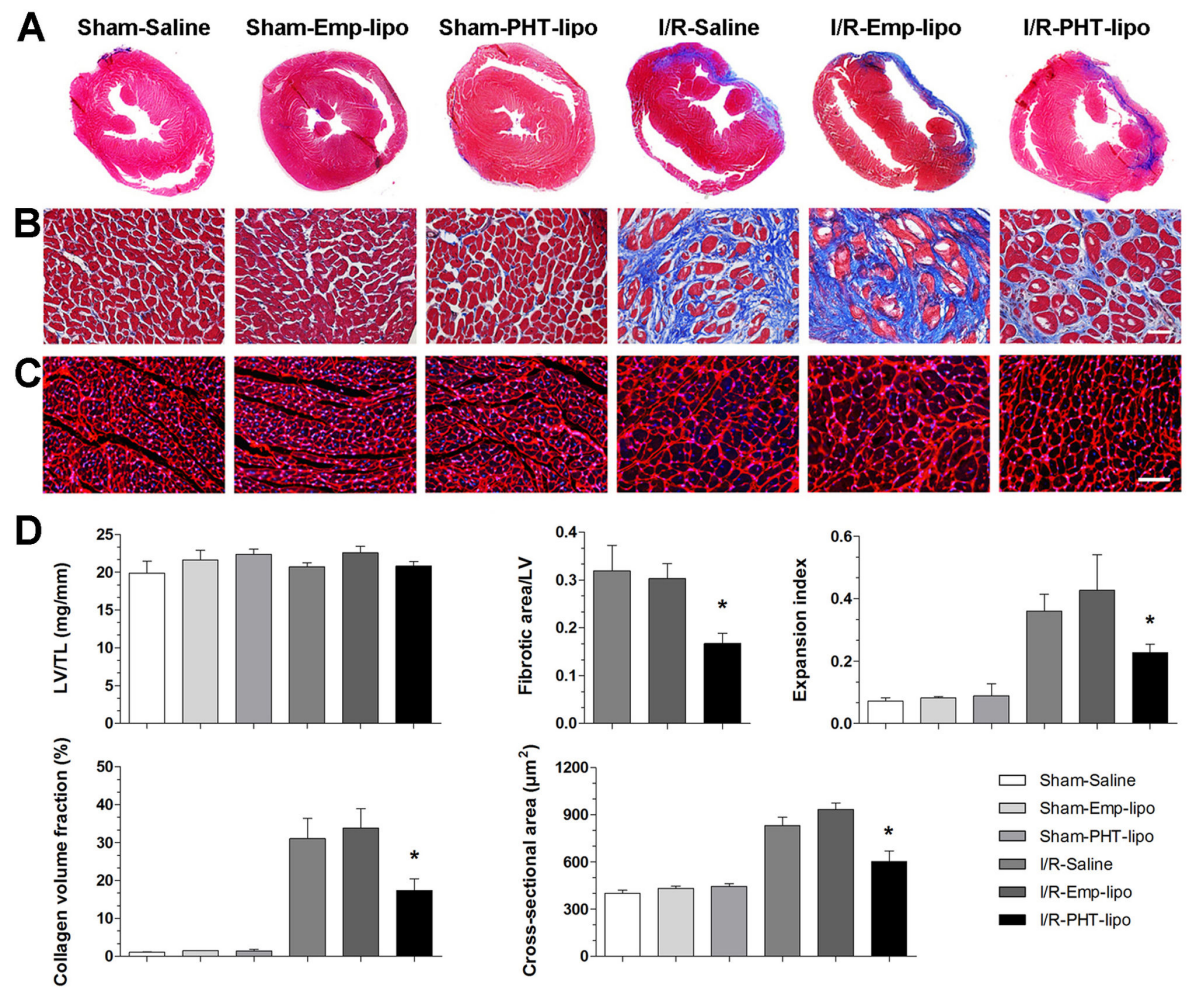
Administration of phenytoin entrapped liposome attenuates pathological post-infarction left ventricular remodeling in rats. (A) Masson's trichrome staining shows the representative gross morphology of heart sections. (B) Collagen deposition in left ventricular free wall/infarct area revealed by Masson’s trichrome staining. Bar=50 µm. (C) TRITC-conjugated wheat germ agglutinin staining to reveal cardiomyocyte cross-sectional area in non-infarcted area. Bar=50 µm. (D) Statistical comparisons of the left ventricular mass to tibial length (LV/TL), fibrotic area to total left ventricular area, expansion index, collagen volume fraction and cardiomyocyte cross-sectional area. *P<0.05 vs. I/R-Emp-lipo group.

**Figure 6:**
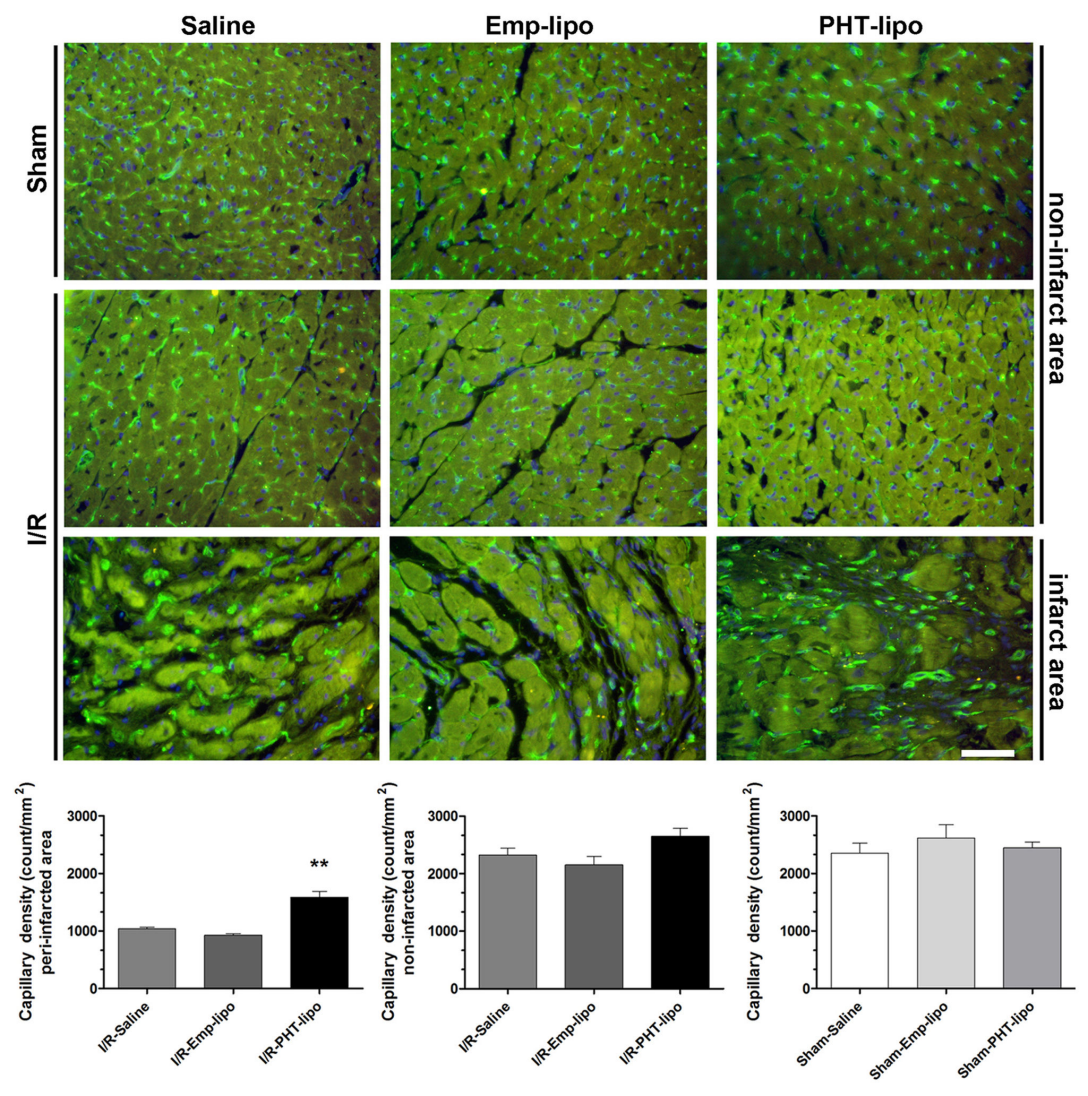
Representative immunofluorescence images of capillary vessels in the infarcted regions. FITC-labeled isolectin B4 (green) was used to detect capillary vessels density. Nuclei were counterstained with DAPI (blue). Phenytoin liposome (PHT-lipo) treatment is associated with increased capillary vessel density in ischemic area. There was no significant difference in capillary density among groups in non-infarcted area. **P<0.01 vs. I/R-Emp-lipo group.

Figure **5** shows the histological examinations on heart sections from animals sacrificed 30 days after I/R injury. PHT-lipo administration could improve post-infarction left ventricular remodeling, as shown by decreased infarct size and left ventricular expansion index, decreased left ventricular fibrotic area, and reduced myocardial cross-sectional area in non-infarcted region. There was no difference in the ratio of left ventricle mass/tibial length (LV/TL) among all groups. As shown in Figure **6**, a significantly increase of isolectin B4 positive capillaries was observed in I/R-PHT-lipo group (*P*<0.01 vs. I/R-Emp-lipo group). In addition, no difference of above mentioned parameters was observed between I/R-Emp-lipo and I/R-Saline groups.

### PHT-lipo improves left ventricular function after myocardial I/R injury

**Figure 7:**
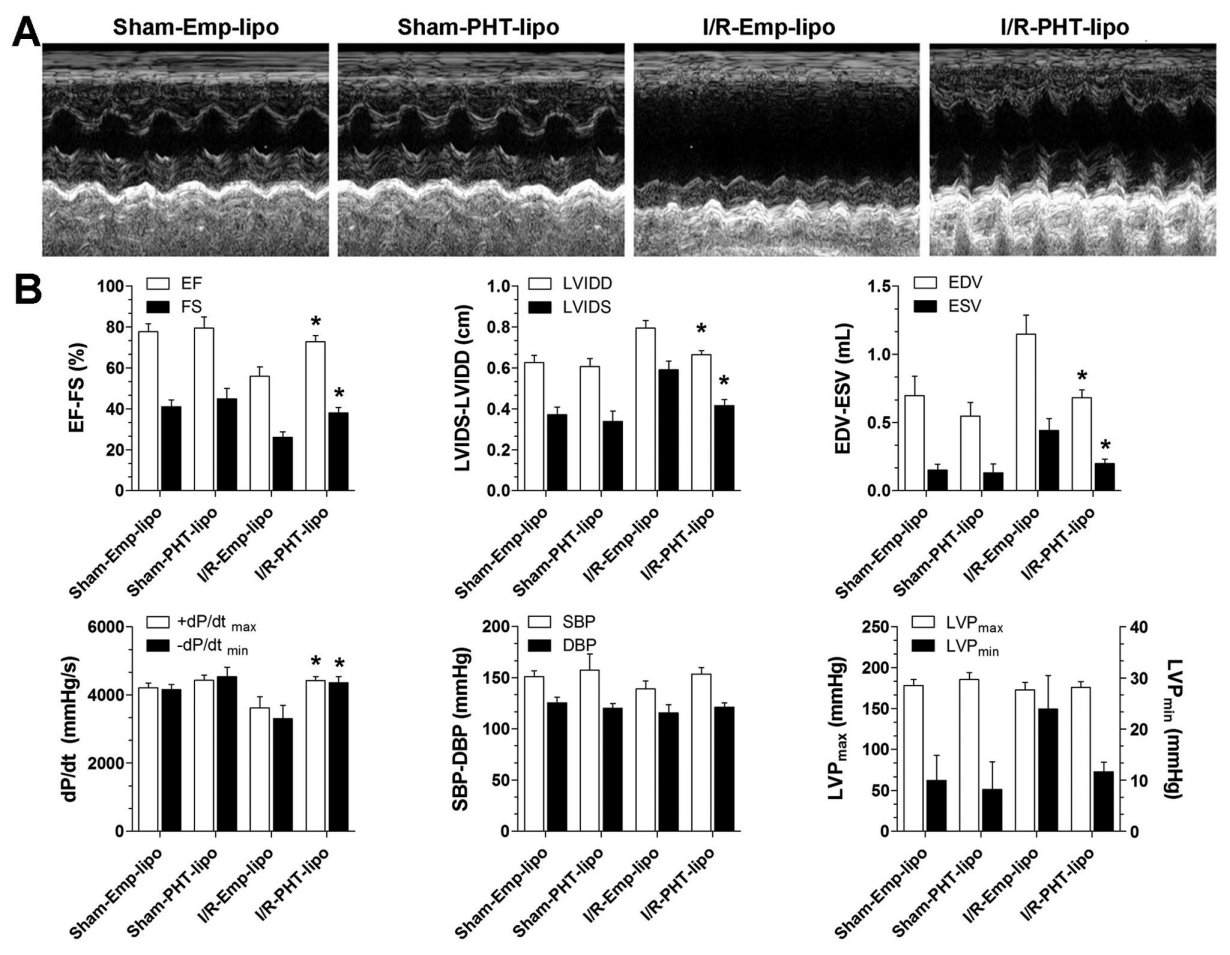
Transthoracic echocardiography and invasive left ventricular hemodynamic analysis. (A) Representative M mode echocardiographic images. (B) Statistical comparisons of echocardiographic and invasive hemodynamic analysis derived parameters. EF, ejection fraction; FS, fractional shortening; LVIDD, left ventricular internal diameter during end diastole; LVIDS, left ventricular internal diameter during end systole; EDV, end-diastolic volume; ESV, end-systolic volume. +*dp*/*dt*_max_, maximal slope of systolic pressure increment; -*dp*/*dt*_min_, maximal slope of diastolic pressure decrease. SBP, systolic blood pressure; DBP, diastolic blood pressure; LVP, left ventricular pressure. *P<0.05 vs. I/R-Emp-lipo group.

30 days after myocardial I/R injuey, PHT-lipo treatment significantly improved left ventricular function compared with Emp-lipo treated group, as determined by transthoracic echocardiography (Figure **7A and 7B**, increased EF and FS; reduced EDV, ESV, LVIDS and LVIDD) (Figure **6B**). In addition, data from invasive hemodynamic measurements also support improved left ventricular systolic (+*dP/dt*_max_) and diastolic (-*dP*/*dt*_min_) functions (Figure **7B**).

## Discussion

The present work using liposome-based and mononuclear phagocyte-targeted delivery of phenytoin, demonstrated that VGSC antagonism during the acute phase after myocardial I/R injury could inhibit the expansion of circulating CD43+ inflammatory monocytes (classical monocytes) and attenuates adverse left ventricular remodeling in a rat model of myocardial I/R injury. In addition, we also showed that VGSC antagonism could suppress LPS-induced M1 polarization and facilitate IL-4-induced M2 polarization in rat peritoneal macrophages. Taken together, these results support the interpretation that modulation of mononuclear phagocyte inflammatory response via liposome entrapped VGSC inhibitor might be a novel approach for reducing myocardial I/R injury.

Accumulating evidence indicates that VGSCs play an important role in regulating phenotype switch of macrophages [19-21]. Here, we demonstrated mRNA expression of several VGSC α and β subunits (NaV1.1, NaV1.3, NaV1.4, NaV1.5, NaV1.6, NaV1.7, NaVX, Scn1b, Scn3b and Scn4b) in rat peritoneal macrophages. Admittedly, their expressions at protein level are not confirmed, due to the lack of commercially available specific antibodies to these subunits. However, VGSC antagonism using PHT, a non-selective inhibitor, suppressed LPS-induced M1 polarization and facilitated M2 marker expression in IL-4-induced alternative activation, providing indirect evidence supporting their functions in macrophage phenotype regulation. In addition, using RNA interference, we recently showed that NaV1.9 knockdown in RAW264.7 macrophage cell line results in anti-inflammatory effect (reduced phagocytosis and migration) [34]. Moreover, our recent preliminary work using purified mouse blood CD11b+ CD45-Ly6G- monocytes also demonstrated the mRNA expression of several VGSC subunits in mouse monocytes (data not shown). There data suggested that VGSC expression in mononuclear phagocyte system might be a common phenomenon involved in their phenotype regulation.

In steady state, recent studies showed that there is a minimal contribution of circulating monocytes to the replenishment of tissue macrophages [35,36]. However, during inflammation, it is generally thought that in mice the Ly6C+ “inflammatory” and Ly6- non-classical monocytes differentiate into M1 (classically activated) and M2 (alternatively activated) macrophages, respectively [37]. In addition, emerging evidence suggested a conversion from inflammatory monocytes to anti-inflammatory M2 type monocytes/macrophages [38]. Currently, rat circulating monocyte subsets are defined by their differential expression of surface maker CD43 [31,39]. A CD43+ subset was found to be similar to the classical subset (for human, CD14++CD16-; for mouse, Ly6C+) and a CD43++ subset was found to be the counterpart to the human and mouse nonclassical subset (for human, CD14+ CD16+ +; for mouse, Ly6C-) [6]. The therapeutic potential of monocyte/macrophage targeting using chemical/drug-loaded, unmodified liposome has been confirmed in recent studies [8,40]. Moreover, clodronate liposome is a well-characterized research tool for mononuclear phagocyte depletion [41,42]. Our pharmacokinetic study showed that PHT-encapsulated liposomes were quickly removed from plasma compared with the free drug after intravenous injections, indicating efficient uptake by mononuclear phagocytes. Subsequent flow cytometry analysis showed that PHT-loaded liposome can partially suppress myocardial I/R injury induced CD43+ inflammatory monocyte expansion. Excessive and prolonged activity of inflammatory monocytes is one of the mechanisms contributing to MI- and I/R injury-induced left ventricular dysfunction. Recently serial studies by Nahrendorf and co-workers have provided ample evidence that mouse Ly6C+ monocytes, the counterpart to rat CD43+ monocytes, is the therapeutic target for many inflammatory diseases [9,10,43-45], especially atherosclerosis and MI. Specifically, Ly6C+ monocytes infiltrate to the ischemia area early after MI (day 1 to 4) and exhibited phagocytic, proteolytic, and pro-inflammatory functions. Whereas Ly6C- monocytes dominate the later phase (from day 5) and attenuated inflammatory response, promoted infarct healing via myofibroblast proliferation, angiogenesis, and maturation of collagen. Monocyte-directed RNAi that inhibits Ly6C+ monocyte improves post-MI healing in atherosclerosis-prone mice [44]. Our results showed that the expansion of inflammatory CD43+ subset dominated on day 3 after myocardial I/R, followed by a gradual decreasing trend. We thus used a three-single-injection protocol on day 0, day 2, and day 4 to cover this time frame in order to target CD43+ monocyte expansion. The results showed this this treatment regimen could efficiently suppress CD43+ pool expansion. Based on previous work by other groups [9,10,43-46], we speculated the counteraction of early inflammatory response may reduce inflammatory monocyte tissue infiltration, contributing to less tissue destruction, and shifting the local microenvironment towards anti-inflammation and presumably facilitating the healing process during the late inflammation. Therefore, our results provide evidence supporting the emerging concept that inflammatory monocyte subset is a therapeutic target for post-infarction heart failure.

In this work, we chose a non-selective VGSC blocker (PHT) because our in vitro studies showed that among the currently recognized 10 α subunits and 4 β VGSC subunits, the mRNA expressions of 7 α and 3 β subunits were demonstrated in rat macrophages. At current stage, it is difficult to dissect the contribution of individual subunits in the regulation of mononuclear phagocyte phenotype. Thus, a non-selective blocking agent would be preferred. Whereas, the relative contribution of individual VGSCs to modulate monocyte/macrophage remains to be elucidated. It has been demonstrated that PHT treatment is associated with enhanced local production of vascular-endothelial growth factor (VEGF) [47], acidic fibroblast growth factor [48], platelet-derived growth factor [49] and TGF-β1 [50], which are all essential for neovascularization. Moreover, compared with Ly6C+ subset, Ly6C- monocytes selectively expressed higher levels of VEGF [43]. Therefore, monocyte/macrophage targeting using PHT-liposome, presumably via an altered local balance between proinflammatory and anti-inflammatory monocyte/macrophage mediated paracrine effect, leads to a more favorable environment to reduce I/R injury and facilitates healing. From the phenomenon we observed, we speculated that monocyte VGSCs is involved in rat blood monocyte homeostasis. Although liposome encapsulated drug has advantages over unformulated drug in terms of lower dose, less drug-related side effect, as well as a targeted effect on mononuclear phagocytes, the difference in formulation will undoubtedly lead to altered biodistribution, especially more distribution in monocytes/macrophages. Therefore, the impact of PHT liposome on monocytes/macrophages-mediated innate immunity needs to be verified.

In conclusion, the present work for the first time demonstrated the therapeutic potential of VGSC antagonism via liposome mediated monocyte/macrophage targeting in acute phase after myocardial I/R injury, which could suppress circulating CD43+ inflammatory monocyte pool expansion, and improves post-infarction left ventricular remodeling. Our results suggest that VGSCs in mononuclear phagocyte system might be a novel target for immunomodulation and treatment of myocardial I/R injury. The exact mechanism by which VGSC inhibition links monocyte subset/macrophage phenotype switch is warranted in future studies.
